# Chirality-Induced Orbital Selectivity through Linear-Orbital Coupling

**DOI:** 10.1021/acs.jpclett.6c01552

**Published:** 2026-07-21

**Authors:** Namgee Cho, James Lim, Martin B. Plenio

**Affiliations:** Institut für Theoretische Physik, 9189 Universität Ulm, Albert-Einstein-Allee 11, D-89081 Ulm, Germany

## Abstract

We present a three-dimensional continuum model of electron transmission through a chiral electrostatic potential and show that it gives rise to chirality-induced orbital selectivity. In this model, electron transmittance depends strongly on the incident orbital angular momentum (OAM) associated with its transverse motion, and the selectivity reverses when the potential’s handedness is inverted. The effect originates from a coupling between axial linear momentum and OAM mediated by the helical spatial dependence of the potential. For DNA-scale geometric parameters, this linear-orbital coupling produces sizable orbital selectivity, which remains robust to static and dynamic disorder, and increases with the length of chiral regions. Although bare spin–orbit coupling in the chiral potential considered here is too weak to generate considerable spin dynamics, spin-OAM correlations in the electrodes allow the same orbital-selective mechanism to induce appreciable spin selectivity. These results identify orbital dynamics as an important contributor to electron transport in chiral systems.

## Graphical abstract

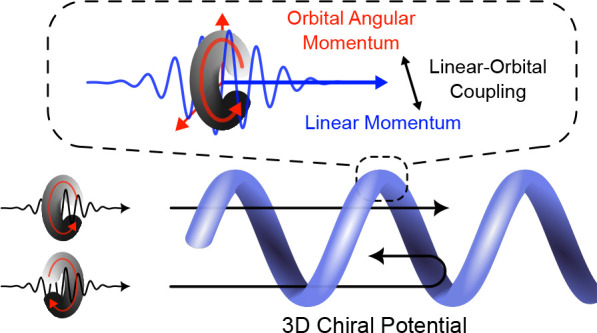

The transfer of electrons through chiral molecules is known to exhibit a pronounced dependence on molecular handedness, exemplified by phenomena such as the chirality-induced spin selectivity (CISS).^1,2^ This effect manifests as substantial enantiospecific differences in electron transmittance, observed in photoemission,^3–6^ magnetoresistance,^7–11^ and magnetic resonance experiments.^12–14^ Despite extensive investigation, the precise microscopic mechanisms underlying CISS remain elusive, as models based solely on spin–orbit coupling (SOC) fail to fully account for the magnitude of the experimentally measured effects.^1^ Theoretical efforts have predominantly relied on simplified electron descriptions, such as low-dimensional tight-binding^15–31^ or one-dimensional continuous-variable models,^32–34^ due to the complexity inherent in fully three-dimensional simulations. These reduced models have incorporated various physical factors, such as electron–electron correlations,^21,26,35^ spin-phonon coupling,^15,23–26^ ^,^ ^29,33,36^ and interfacial effects,^26,27,31^ ^,^ ^32,37^ which have been shown to enhance chirality-dependent electron dynamics. Alternatively, it has been proposed that a substrate with strong SOC generates spin-OAM-correlated electrons, and that the chiral molecule may subsequently filter their OAM states, indirectly inducing spin selectivity via the correlations.^18,28^ The potential relevance of OAM in photoelectron scattering has also been studied.^38^ However, although previous theoretical studies have suggested the potential importance of orbital angular momentum in CISS, a microscopic mechanism underlying orbital selectivity has not yet been proposed, and the robustness of orbital selectivity under various physical conditions, including variations in the electronic potential landscape and the presence of disorder in chiral systems, remains unexplored.

In this work, we investigate a three-dimensional (3D) continuous-variable model of an electron propagating through a chiral electrostatic potential. The transverse confinement defines local orbital-angular-momentum states around the helical centerline, and transmission through the chiral region depends strongly on the incident OAM state. Moreover, the orbital polarization changes sign when the handedness of the chiral potential is inverted. In our model, chirality-induced orbital selectivity originates from a helical coupling between axial linear momentum and OAM of an electron, rather than OAM filtering or anisotropic hopping through atomic orbitals in a reduced-dimensional description as in refs 18 and 28 (see the SI for details). Our CIOS mechanism is analogous in spirit to CISS induced by delocalized phonon modes.^33^ However, crucially, the linear-orbital coupling, which originates from the transverse electron motion and is responsible for the CIOS effect, is significantly stronger than the spin-phonon coupling under physically reasonable parameter regimes. We demonstrate that the CIOS effect increases with the length of the chiral region and remains robust against static disorder in its internal potential structure. We remark that while bare spin–orbit coupling inside the chiral potential is too weak to induce appreciable spin dynamics in our 3D model, spin-OAM correlations, present prior to transmission through the chiral potential, allow the CIOS mechanism to induce the CISS effect. These results suggest that CIOS may play an important role in enantiospecific electron transmission in chiral systems.

We consider a model in three spatial dimensions of an electron propagating through a chiral potential whose long axis coincides with the *z*-axis and is defined in the region 0 ≤ *z* ≤ *L*. The Hamiltonian is given by

1 $$H=\frac{1}{2m_{e}}\left(p_{x}^{2}+p_{y}^{2}+p_{z}^{2}\right)+\frac{m_{e}\omega^{2}}{2}{\left(x-R\cos\left(\frac{z}{P}\right)\right)}^{2}+\frac{m_{e}\omega^{2}}{2}{\left(y-R\sin\left(\frac{z}{P}\right)\right)}^{2}$$

where the equilibrium position of the harmonic potential is displaced in the *xy*-plane as a function of *z*, encoding the chirality of the chiral potential, as schematically shown in Figure 1a. To reduce the computational cost of simulating our 3D model, we consider a polaron transformation implemented via a unitary displacement operator conditioned on *z*

2 $$U=e^{iR\prime e^{-\mathrm{iz}/P}\left(c^{†}-d\right)+iR\prime e^{\mathrm{iz}/P}\left(c-d^{†}\right)}$$

with $R^{\prime }=\sqrt{m_{e}\omega /4\hbar }R$ and a set of independent bosonic operators defined as c= $\sqrt{m_{e}\omega /4\hbar }$ $\left(y+\mathrm{ix}\;+\left({\mathrm{ip}}_{y}-p_{x}\right)/m_{e}\omega \right)$ , and d= $\sqrt{m_{e}\omega /4\hbar }(y-\mathrm{ix}\;+$ $\left({\mathrm{ip}}_{y}+p_{x}\right)/m_{e}\omega )$ , satisfying the canonical commutation relations [*c*, *c* ^†^] = [*d*, *d* ^†^] = 1 and [*c*, *d* ^†^] = 0 (see the SI). Then *H*′ = *U* ^†^ *HU* is given by

3 $$H^{\prime }=\hbar \omega \left(c^{†}c+d^{†}d+1\right)+\frac{{\left(p_{z}+i\beta \left(e^{\mathrm{iz}/P}\left(c-d^{†}\right)-e^{-\mathrm{iz}/P}\left(c^{†}-d\right)\right)\right)}^{2}}{2m_{e}}$$

with $\beta =\left(R/2P\right)\sqrt{\hbar m_{e}\omega }$ , where the kinetic energy along the *z*-direction acquires a chiral contribution. In this work, we consider the parameters of DNA, specifically, the radius R = 1.0 nm and the pitch 2*πP* = 3.4 nm, along with the free-electron mass *m*_*e*_. The frequency ω of the harmonic potential is chosen such that ℏω ∈ [0.05, 5] eV, ensuring that the size of the ground-state wavepacket in the *xy*-plane defined by its standard deviation satisfies $\sqrt{\hbar /\left(2m_{e}\omega \right)}\in$ [1,10]Å . These parameters lead to ⟨*p*_*z*_⟩β/2*m*_*e*_ ∈ [0.15, 1.5] eV and β^2^/2*m*_*e*_ ∈ [0.02, 2] eV for a reference value of ⟨*p*_*z*_^2^⟩/2*m*_*e*_ = 1 eV.

**Figure 1:**
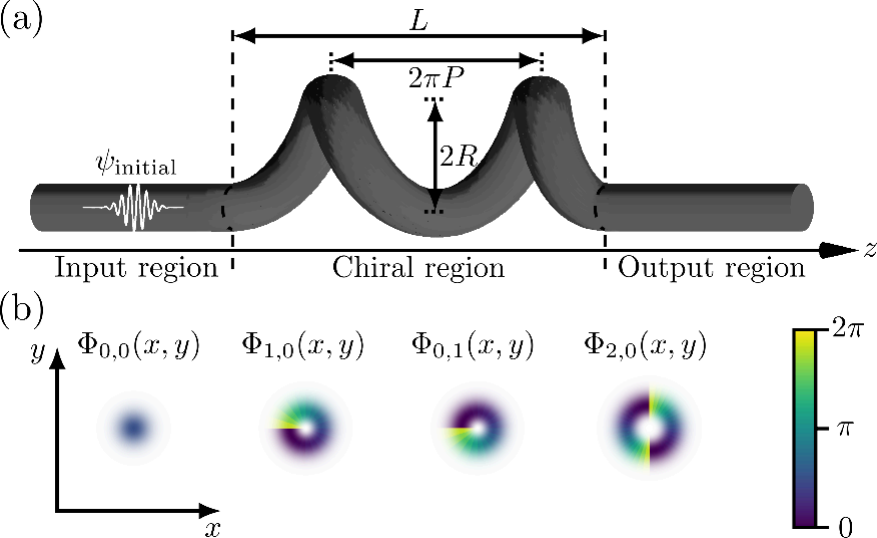
(a) Schematic representation of a 3D model for electron transfer through input-chiral-output regions. The input and output regions are achiral and elongated along the *z*-axis, and are continuously connected to the chiral region, which is parametrized by radius *R*, pitch 2*πP*, and length *L*. For each *z*, a 2D harmonic potential in the *xy*-plane is considered. (b) Phase distributions of OAM states Φ_*n*_*c*_,*n*_*d*__(*x, y*) in the *xy*-plane. The amplitudes of the OAM states are used as an opacity factor, so that the phase is visualized only where the amplitudes are sufficiently large.

In the polaron picture, the *z*-component of the OAM operator is defined as *L*_*z*_ = *xp*_*y*_ – *yp*_*x*_ = ℏ­(*c* ^†^ *c* – *d* ^†^ *d*), where the eigenstates of *L*_*z*_ with OAM ℏ­(*n*_*c*_ – *n*_*d*_) are described by the composite eigenstates |*n*_*c*_, *n*_*d*_⟩ of the harmonic modes *c* and *d*, where *n*_*c*_ and *n*_*d*_ are non-negative integers. Here, positive and negative OAM correspond, respectively, to right- and left-circular motion of the electron about the *z*-axis. In the original frame without the polaron transformation, the *L*_*z*_ operator describes the OAM of an electron with respect to a local coordinate system whose origin follows the chiral path (*x*, *y*) = (*R* cos­(*z/P*), *R* sin­(*z/P*)) parametrized by *z*. In Figure 1b, the phase distributions of the OAM states in the *xy*-plane at a fixed *z* are shown (i.e., Φ_*n*_*c*_,*n*_*d*__(*x, y*) = ⟨*x*, *y*|*n*_*c*_, *n*_*d*_⟩ in the position basis |*x*, *y*⟩), demonstrating that the Φ_1,0_ and Φ_0,1_ states, which carry nonzero OAM of ℏ and −ℏ, respectively, exhibit opposite chiralities in their phase distributions, in contrast to the Φ_0,0_ state with zero OAM, which shows an achiral phase distribution.

We note that the chiral term in the kinetic energy induces the interaction between linear and orbital angular momenta, such that when the OAM decreases by ℏ via *c* – *d* ^†^, the linear momentum increases by ℏ/*P* via e^*iz*/*P*^, and vice versa. To demonstrate that this linear-orbital coupling can induce transmittance depending on the initial OAM states, we consider achiral input and output regions in the laboratory frame where the equilibrium position of the harmonic potential in the *xy*-plane is independent of *z*. These regions are smoothly connected to the chiral potential in eq 1, as shown in Figure 1a, and serve as a simple model of electrodes coupled to a chiral molecule.^7–11^ The Hamiltonian for the input and output regions in the polaron picture is given by eq 3 with β = 0. As shown in Figure 1a, we consider an initial wave packet localized in the input region, i.e., ψ_initial_ ∝ Φ_*n*_*c*_,*n*_*d*__(*x, y*)­e^–(*z*–*z*_0_)^2^/(2Δ_*z*_^2^)+*ik*_0_ *z*^, carrying linear momentum ℏ*k*_0_ and OAM ℏ­(*n*_*c*_ – *n*_*d*_) about the *z*-axis. We set the initial kinetic energy to KE_0_ = (ℏ*k*_0_)^2^/2*m*_*e*_ = 1 eV, and the width to Δ_*z*_ = 4 nm. The propagation of the electron wave packet is numerically simulated using the finite-difference method.

We proceed to show that electron scattering within the chiral region is governed by (i) energy conservation and (ii) a coupled change in linear and orbital angular momenta which, together, underpin the CIOS effect. In Figure 2, we consider two distinct initial states with (*n*_*c*_, *n*_*d*_) = (1, 0) or (0, 1), both having the same linear momentum ℏ*k*_0_ in the *z*-direction. To clarify the mechanism of chirality-induced orbital selectivity, we examine a weak-coupling regime in which the chiral coupling strength β based on the DNA parameters is reduced by 2 orders of magnitude (i.e., β = β_DNA_/100); simulation results using the full chiral coupling strength will be presented in Figure 3. In the weak-coupling regime, the electron energy is approximately given by *E* ≈ ℏω­(*n*_*c*_ + *n*_*d*_ + 1) + (ℏ*k*_0_)^2^/2*m*_*e*_, implying that the two initial states with opposite OAM have the same energy. Starting from the initial OAM state |1, 0⟩, a transition to |0, 0⟩ (or |2, 0⟩) results in an increase (or decrease) of the linear momentum from ℏ*k*_0_ to ℏ*k*_1_ due to energy conservation, i.e., (ℏ^2^/2*m*_*e*_)­(*k*_1_^2^ – *k*_0_^2^) = ℏω (or (ℏ^2^/2*m*_*e*_)­(*k*_1_^2^ – *k*_0_^2^) = – ℏω). This transition is mediated by the chiral coupling term proportional to *c*e^*iz/P*^ (or *c* ^†^e^–*iz/P*^), which increases (or decreases) the linear momentum by ℏ/*P*, i.e., *k*_1_ – *k*_0_ = 1/*P* (or *k*_1_ – *k*_0_ = −1/*P*). For the initial kinetic energy of KE_0_ = (ℏ*k*_0_)^2^/2*m*_*e*_ = 1 eV and the pitch of 2*πP* = 3.4 nm, both conditions are fulfilled when ℏω ≈ 0.85 eV (or 0.59 eV). For the other initial OAM state |0, 1⟩, the two conditions are satisfied only for the transition |0, 1⟩ → |1, 1⟩. Since this transition is induced by the chiral coupling ∝ ⟨1|*c* ^†^|0⟩, which is weaker than the coupling ∝ ⟨2|*c* ^†^|1⟩ responsible for the transition |1, 0⟩ → |2, 0⟩, the latter transition occurs with a higher probability. In contrast, transitions induced by the other chiral coupling term *d*e^–*iz*/*P*^ (or *d* ^†^e^*iz/P*^), such as |0, 1⟩ → |0, 0⟩ (or |0, 1⟩ → |0, 2⟩), cannot simultaneously satisfy energy and momentum conservation because the chiral nature of the coupling modifies the linear momentum in a way that violates energy conservation. Therefore, electron dynamics within the chiral region depends on the initial OAM state.

**Figure 2:**
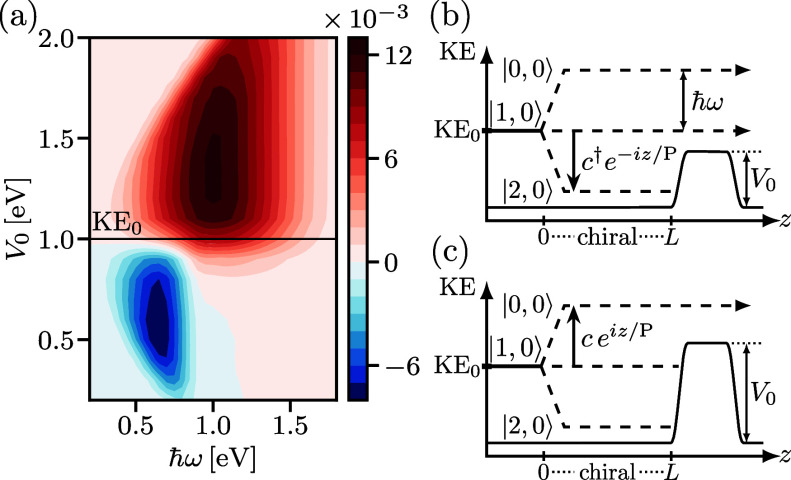
(a) Transmittance difference *ΔT* = *T*_1,0_ – *T*_0,1_ between the initial OAM states |1, 0⟩ and |0, 1⟩ as a function of the barrier height *V*_0_ and the frequency ℏω of the transverse harmonic potential in the weak-coupling regime (β = β_DNA_/100). The initial kinetic energy (KE) in the *z*-direction, KE_0_ = 1 eV, is indicated by the solid line. The length of the chiral region is taken as *L* = 2*πP*. For the initial state |1, 0⟩: (b) Schematic of the effect of transitions between OAM states when *V*_0_ < KE_0_. The transition |1, 0⟩ → |2, 0⟩, induced by a linear-orbital coupling ∝ *c* ^†^e^–*iz*/*P*^, reduces the kinetic energy along *z* and causes reflection at a barrier, which results in a lower transmittance than the other initial state |0, 1⟩ (*ΔT* < 0). (c) Schematic of the effect of transitions when *V*_0_ > KE_0_. The transition |1, 0⟩ → |0, 0⟩, induced by another linear-orbital coupling ∝ *c*e^*iz/P*^, enables electron transmission through a barrier, which results in a higher transmittance than the other initial state |0, 1⟩ (*ΔT* > 0).

**Figure 3:**
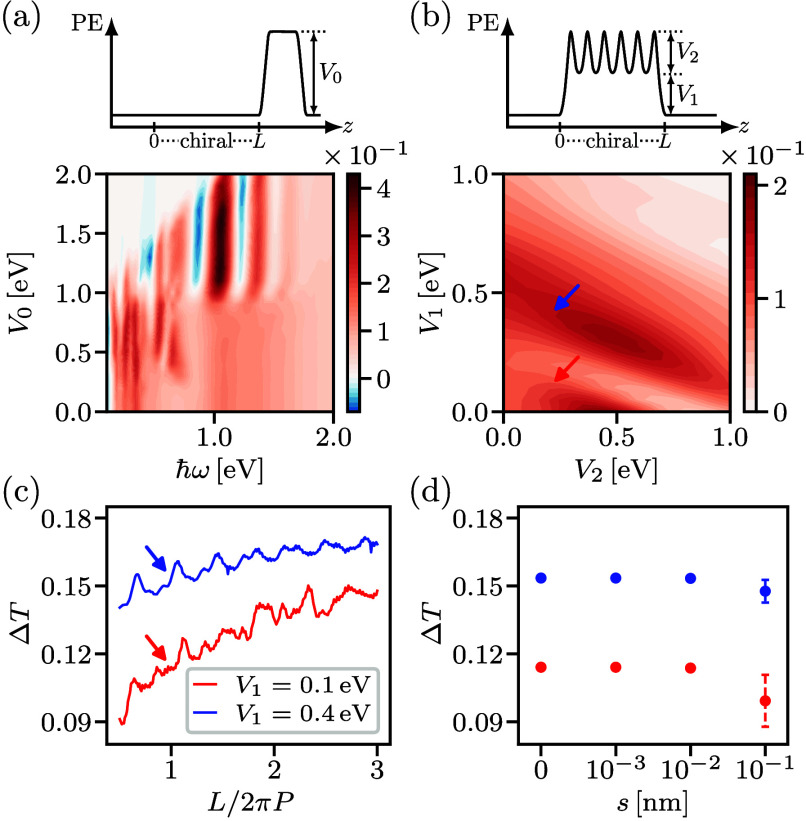
(a) *ΔT* as a function of *V*_0_ and ω in the strong-coupling regime (β = β_DNA_), with *L* = 2*πP*, as in Figure 2a. (b) *ΔT* as a function of *V*_1_ and *V*_2_ in the lattice-potential model, with β = β_DNA_ and *L* = 2*πP*. (c) *ΔT* as a function of *L* for two cases, (*V*_1_, *V*_2_) = (0.1, 0.2)­eV and (0.4, 0.2)­eV, marked by arrows in (b). (d) *ΔT* as a function of the standard deviation *s* of static disorder in the lattice-potential structure, with *L* = 2*πP* and β = β_DNA_, for the two cases marked in (c). Corresponding orbital polarizations are shown in Figure SI.4.

To demonstrate that different electron dynamics, depending on the initial OAM state, can result in distinct transmission probabilities through the chiral region, in Figure 2, we consider a rectangular potential barrier along the *z*-direction, located after the chiral region, with a controlled barrier height *V*_0_ and a fixed width of 1 nm. Even if the barrier is placed inside the chiral region, the transmission remains dependent on the initial OAM state (not shown). We examine the difference in transmittance of the two initial states, i.e., *ΔT* = *T*_1,0_ – *T*_0,1_, as a function of the barrier height *V*_0_ and the frequency ω of the harmonic potential in the *xy*-plane, where *T*_*n*_*c*_,*n*_*d*__ denotes the total population of the electron wave packet in the output region after scattering through the chiral region, given the initial OAM state |*n*_*c*_, *n*_*d*_⟩. For barrier heights lower than the initial kinetic energy, i.e., *V*_0_ < 1.0 eV, a negative transmittance difference (*ΔT* < 0) appears around ℏω ≈ 0.65 eV, because the transition from |1, 0⟩ to |2, 0⟩ lowers the kinetic energy, causing the electron to be reflected by the barrier, and thus reducing the transmittance of the initial OAM state |1, 0⟩, as illustrated in Figure 2b. The transition from the other initial state |0, 1⟩ to |1, 1⟩ likewise results in reflection by the barrier, but occurs with a lower probability. These transient electronic dynamics can be tracked in simulations (see Figure SI.1 in the SI). For barrier heights higher than the initial kinetic energy, i.e., *V*_0_ > 1.0 eV, the transmittance difference exhibits a broad positive peak (*ΔT* > 0) centered around ℏω ≈ 1 eV. In this regime, the initial kinetic energy is insufficient for direct transmission, making the transition from |1, 0⟩ to |0, 0⟩ crucial, as schematically shown in Figure 2c. We note that the extrema in Figure 2 occur close to, but not exactly at, the values expected from the bare matching condition, namely ℏω ≈ 0.85 eV (or ℏω ≈ 0.59 eV). This deviation reflects the ω-dependence of the coupling amplitude $\beta =\left(R/2P\right)\sqrt{\hbar m_{e}\omega }$ . We verified this by evaluating the transmittance difference for a fixed β at a reference frequency ω_0_ with ℏω_0_ = 1 eV. This yields a positive peak centered at ℏω ≈ 0.85 eV, and a negative peak at ℏω ≈ 0.59 eV (See Figure SI.2 in the SI). These results indicate that the coupling strength β and the associated transition matrix element, which increase with ω, are responsible for shifting the peak from 0.85 to 1.0 eV (or from 0.59 to 0.65 eV).

In Figure 3a, the transmittance difference *ΔT* is shown as a function of *V*_0_ and ω, similar to Figure 2a, but now for the linear-orbital coupling based on DNA-scale parameters (β = β_DNA_). In this strong-coupling regime, the electron’s energy can no longer be expressed simply as the sum of the OAM energy and the kinetic energy in the *z*-direction, and thus the analysis used in the weak-coupling regime of Figure 2 is no longer applicable. However, the strong coupling gives rise to richer OAM dynamics and enhanced orbital selectivity. In contrast to the weak-coupling case, orbital selectivity with nonzero *ΔT* appears even in the absence of the potential barrier (*V*_0_ = 0), due to the OAM-dependent reflection at the interface between the input and chiral regions (see Figure SI.3 in the SI). Notably, *ΔT* in Figure 3a is one to 2 orders of magnitude larger than in the weak-coupling case in Figure 2a, over a broad range of *V*_0_ and ω. This enhanced orbital selectivity still originates from the coupled change in linear and orbital angular momenta, as can be seen from the fact that *ΔT* vanishes when the factors e^± *iz/P*^ in eq 3, which shift the linear momentum conditioned on the OAM transitions, are removed. We find that for *V*_0_ ≲ KE_0_ = 1 eV, *ΔT* remains positive, whereas for *V*_0_ ≳ 1 eV, *ΔT* can take both positive and negative values. For the data shown in Figure 3a, the orbital polarization, defined as the transmittance difference *ΔT* divided by the total transmission probability, reaches several tens of percent, up to 80% (see Figure SI.4 in the SI). This high orbital polarization arises primarily from the initial-OAM-dependent transition to |0, 0⟩, analogous to the weak-coupling case shown in Figure 2c, as confirmed numerically by the OAM spectrum of the transmitted wave packet (see Figure SI.5 in the SI). When the handedness of the 3D chiral potential is reversed, the sign of *ΔT* and the orbital polarization also reverse, while the line shapes in Figure 3a remain unchanged.

So far, we have not considered any internal potential structure within the chiral region. We now introduce a lattice-like potential as a function of *z* inside the chiral region, parametrized by an offset *V*_1_ and the height *V*_2_ of multiple Gaussian potentials, as schematically illustrated in Figure 3b, with fixed ℏω = 0.5 eV. For a single chiral turn with *L* = 2*πP*, we place six Gaussian potentials with uniform spacing and each of the form e^–(*z*–*z*_*i*_)^2^/2δ^2^^, centered at *z* = *z*_*i*_, with width δ = *P*/5. As shown in Figure 3b, positive *ΔT* on the order of 10^–1^ emerges over a broad range of *V*_1_ and *V*_2_. For the two representative points indicated by arrows in Figure 3b, the dependence of *ΔT* on the length *L* of the chiral region is shown in Figure 3c. Here, *L* increases continuously, so the final Gaussian potential at the boundary between the chiral and output regions may not be fully contained within the chiral region. In the simulations, this final Gaussian potential is smoothly suppressed using a smooth step function to ensure a continuous transition into the output region. Notably, *ΔT* gradually increases with the number of chiral turns, from one to three. This length dependence is associated with the presence of the multiple Gaussian potentials, as *ΔT* shows negligible length dependence when *V*_2_ = 0 (see Figure SI.6 in the SI). These results indicate that the orbital selectivity arises not only at the interfaces between the chiral and input/output regions, but also within the chiral region itself, and that this length dependence occurs even in the absence of electronic dephasing noise.

In Figure 3d, we examine the effect of static disorder in the lattice potential structure for the cases marked by arrows in Figure 3c, with *L* = 2*πP*. Starting from the positions of the six Gaussian potentials with uniform spacing, as considered in Figures 3b and c, the position of each Gaussian is independently shifted, with each shift randomly generated from a zero-mean Gaussian distribution with a controlled standard deviation *s*. As shown in Figure 3d, the magnitude of *ΔT* remains essentially unchanged for *s* up to 10^–2^ nm and shows only a minor change at 10^–1^ nm, covering a broad range of molecular deformation scales induced by phonon motion. Additionally, the CIOS effect remains robust under dynamic disorder, in which the amplitudes of the Gaussian potentials are time-dependent and the electron energy is consequently not conserved (see the SI). These results demonstrate that the orbital selectivity observed in our work is robust against structural disorder in the lattice potential of the chiral region.

In this work, we have demonstrated that a 3D chiral potential with physically reasonable parameters can induce significant orbital selectivity, where the electron transmittance depends on the initial orbital angular momentum state. While the model in the main text assumes a harmonic confinement potential, qualitatively similar results are obtained for anharmonic confinement (see the SI), highlighting the generality of the CIOS effect. Similar CIOS effects are also observed in tight-binding models based on atomic orbitals, and these results will be reported in a forthcoming paper. By employing a wave packet method, which enables monitoring of transient electron dynamics during scattering inside the chiral region, we have clarified a CIOS mechanism in terms of energy and momentum conservation. While we have considered two specific initial OAM states, |1, 0⟩ and |0, 1⟩, and examined their difference in transmittance in the main text, similar CIOS effects are observed for other initial OAM states (see Figure SI.9 in the SI).

For the parameters considered in Figures 2 and 3, we found that the bare spin–orbit coupling *H*_SOC_ = ℏ­(2*m*_*e*_ *c*)^−2^ **σ** · (∇*W* × **p**) induced by the chiral potential energy *W* in eq 1 results in negligible spin dynamics, where **σ** and **p** denote the Pauli spin operator and the electron’s momentum operator, respectively. However, when the electron’s OAM and spin states are correlated prior to transmission through the chiral region, orbital selectivity can give rise to spin selectivity. In the SI, we introduce spin–orbit interaction at the interface between the input and chiral regions to account for the finite SOC of the electrodes. When electrons with random spin and zero OAM enter the nonchiral SOC region, spin flips are accompanied by changes in OAM to conserve the total angular momentum about the long axis of the input region (i.e., |0, 0, ↑⟩ → |1, 0, ↓⟩ and |0, 0, ↓⟩ → |0, 1, ↑⟩). Since OAM states exhibit different transmission probabilities through the chiral region, the spin-OAM correlations lead to both spin and orbital selectivity (see the SI for details).

The chiral terms in eq 1, i.e., those proportional to *x* cos­(*z/P*) and *y* sin­(*z/P*), are analogous to the spin-phonon coupling of ref 33, originating from fluctuations of the spin–orbit coupling of a chiral molecule induced by delocalized phonon motion. The spin-phonon interaction of ref 33 was modeled by σ_SOC_ *A* sin­(2*πs*/λ) where σ_SOC_ is a spin operator, *A* is the amplitude of a harmonic phonon mode with wavelength λ, and *s* is a coordinate describing the electron’s motion along a chiral one-dimensional path. However, there are two key differences. First, in the spin-phonon coupling model, spin selectivity is induced by delocalized phonon modes that typically have low vibrational frequencies (≲ 0.01 eV) that are one to 2 orders of magnitude smaller than the energy quanta ℏω associated with orbital angular momentum states. Such low phonon frequencies may lead to electron transmittance that is less sensitive to the barrier height than the orbital selectivity present in this work. Second, the spin-phonon coupling strength is expected to be weaker than the bare spin–orbit interaction, as the spin-phonon coupling arises as a perturbation of the spin–orbit coupling, suggesting that it is likely to be weak in realistic systems. In contrast, the linear-orbital coupling β, which induces orbital selectivity, has a notable magnitude under physically reasonable parameters. This makes orbital selectivity potentially more relevant and effective than the spin-phonon mechanism in influencing electron transport in chiral systems.

To clarify the relevance of the CIOS mechanism in CISS experiments, a detailed microscopic description of both the chiral molecules and the electrodes is required, for example using tight-binding models with parameters derived from first-principles calculations. Such a framework may enable quantitative comparison with experimental observations of CISS effects and thereby provide a way to assess the viability of the CIOS mechanism. More direct verification would require experimental schemes capable of resolving the OAM states of electrons transmitted through chiral molecules. One possible route is via photoelectron-based experiments. For example, one could analyze how electron OAM and spin polarizations contribute to the diffraction patterns produced by a grating, for which OAM-dependent diffraction has been demonstrated for electron vortex beams,^39–41^ or to the scattering patterns measured by Mott polarimetry.^4,5,38,42^

Our results demonstrate the critical role of orbital angular momentum dynamics and chirality-induced orbital selectivity in enantiospecific electron transmission. These findings complement and extend the recent advances in orbitronics, where chiral materials and crystals are being recognized as promising platforms to generate and control orbital angular momentum for novel transport and device functionalities.^43–45^

## Supplementary Material



## Notes

Polaron transformation of a continuous-variable Hamiltonian with a harmonic confinement potential, summary of differences from previous CIOS studies, simulation results based on continuous-variable models with anharmonic confinement, and the harmonic model with finite bias, with time-dependent potentials, and with spin-OAM-correlated input states (PDF)
