# Supplementary material for: Chirality-Induced Orbital Selectivity through Linear-Orbital Coupling
Source: J Phys Chem Lett. 2026 Jul 21;17(30):8535–40. doi: 10.1021/acs.jpclett.6c01552 (PMC13430687; doi:10.1021/acs.jpclett.6c01552)
Supplement: Supplementary file 1 [file jz6c01552_si_001.pdf]

Supporting Information for: Chirality-Induced Orbital Selectivity through Linear-Orbital Coupling

Namgee Cho,[1] James Lim,[1] and Martin B. Plenio[1,*]

[1] *Institut für Theoretische Physik, Albert-Einstein-Allee 11, Universität Ulm, D-89081 Ulm, Germany*

[*] martin.plenio@uni-ulm.de

I. POLARON TRANSFORMATION

In the main text, we consider the Hamiltonian of the form

$$H = \frac{1}{2m_e}(p_x^2 + p_y^2 + p_z^2) + \frac{m_e\omega^2}{2}\left(x - R\cos(z/P)\right)^2 + \frac{m_e\omega^2}{2}\left(y - R\sin(z/P)\right)^2, \tag{SI.1}$$

where the equilibrium position of the harmonic potential is displaced in the xy-plane, as a function of z. The unitary operator for the polaron transformation, defined in the main text, can be re-expressed as

$$U = \exp\left(R'' \cos(z/P)(a^\dagger - a) + R'' \sin(z/P)(b^\dagger - b)\right), \tag{SI.2}$$

with $R'' = \sqrt{m_e\omega/2\hbar}R$, where $a = \sqrt{m_e\omega/2\hbar}(x + ip_x/m_e\omega)$ and $b = \sqrt{m_e\omega/2\hbar}(y + ip_y/m_e\omega)$ denote, respectively, the annihilation operators of the harmonic oscillators in the x- and y-directions. The unitary operator converts the Hamiltonian H into

$$H' = U^\dagger H U \tag{SI.3}$$

$$= \frac{1}{2m_e}(p_x^2 + p_y^2) + \frac{m_e\omega^2}{2}(x^2 + y^2) + \frac{1}{2m_e}\left(p_z + \frac{R}{P}\sin(z/P)p_x - \frac{R}{P}\cos(z/P)p_y\right)^2, \tag{SI.4}$$

$$= \hbar\omega(a^\dagger a + b^\dagger b + 1) + \frac{\left(p_z + i\beta'\left(\sin(z/P)(a^\dagger - a) - \cos(z/P)(b^\dagger - b)\right)\right)^2}{2m_e}, \tag{SI.5}$$

with $\beta' = (R/P)\sqrt{\hbar m_e\omega/2}$, where the harmonic potential in the xy-plane becomes achiral, while the kinetic energy along the z-direction acquires a chiral contribution. A similar polaron transformation has been shown to decrease the computational cost of spin-boson models, where the equilibrium positions of harmonic oscillators are shifted depending on spin states. We find that our polaron transformation likewise reduces the computational cost for simulating our 3D model.

In the polaron picture, the OAM operator in the z-direction is given by $L_z = xp_y - yp_x = i\hbar(ab^\dagger - a^\dagger b)$. To consider the OAM eigenstates of L_z explicitly, we introduce in the main text a new set of independent bosonic operators defined as $c = (b + ia)/\sqrt{2}$ and $d = (b - ia)/\sqrt{2}$, satisfying the canonical commutation relations $[c, c^\dagger] = [d, d^\dagger] = 1$ and $[c, d^\dagger] = 0$. The OAM operator is then expressed as $L_z = \hbar(c^\dagger c - d^\dagger d)$. The Hamiltonian H' in the polaron picture can also be expressed in terms of the new mode operators c and d

$$H' = \hbar\omega(c^\dagger c + d^\dagger d + 1) + \frac{\left(p_z + i\beta(\mathrm{e}^{iz/P}(c - d^\dagger) - \mathrm{e}^{-iz/P}(c^\dagger - d))\right)^2}{2m_e}, \tag{SI.6}$$

with $\beta = \beta'/\sqrt{2}$.

II. DIFFERENCES FROM PREVIOUS CIOS STUDIES

In the following, we would like to draw attention to and discuss important differences of our model compared to those reported in earlier works, notably Refs. [1, 2].

Underlying physical mechanisms – In Refs. [1, 2], tight-binding models for CIOS were investigated numerically, but the underlying physical mechanisms that give rise to the selectivity were not examined in detail. Instead, these studies primarily focus on the emergence of spin selectivity arising from the interplay of CIOS and the correlations between OAM and spin states induced by strong SOC in a substrate. In contrast, our work identifies the mechanism underlying CIOS as originating from the combination of (i) a coupled change in the OAM and linear momentum along the z-direction due to the chirality of the scattering potential, and (ii) the principles of energy and momentum conservation.

Hilbert space and energetic structure – In Ref. [2], a tight-binding model in one spatial dimension with three sites per chiral turn was studied. Each site was modeled by three atomic p-orbitals of identical energy, giving rise to three fully degenerate OAM states with OAM limited to $+\hbar$, 0, and $-\hbar$. In contrast, our 3D model features electron OAM states $|n_c, n_d\rangle$ (n_c and n_d are non-negative integers) that are only partially degenerate, i.e., $\hbar\omega(c^\dagger c + d^\dagger d)|n_c, n_d\rangle = \hbar\omega(n_c + n_d)|n_c, n_d\rangle$, and allow for unbounded OAM $\hbar(n_c - n_d)$. In the presence of strong coupling that induces transitions between OAM states, restricting the OAM Hilbert space to only $+\hbar$, 0, and $-\hbar$ may not accurately capture the electronic dynamics.

Because of energy conservation, the full degeneracy of the OAM states, as assumed in Ref. [2], can significantly influence the CIOS effect. In our model, a coupled change in the OAM and linear momentum along the z-direction is crucial. Due to the energy differences of OAM states, this may lead to changes in the kinetic energy in the z-direction, which in turn affects transmittance through the chiral region containing potential barriers, as detailed in the main text. If the OAM energy term $\hbar\omega(c^\dagger c + d^\dagger d)$ is removed from our simulations, making the OAM states fully degenerate, a change in OAM cannot alter the kinetic energy due to energy conservation.

In Fig. SI.7, we consider a rectangular potential barrier located after a chiral region, as in Figs. 2 and 3(a) of the main text, and show how omitting the OAM energy term $\hbar\omega(c^\dagger c + d^\dagger d)$ affects the transmittance difference $\Delta T = T_{1,0} - T_{0,1}$ in simulations.

In the weak-coupling regime ($\beta = \beta_{\mathrm{DNA}}/100$), the transmittance difference ΔT vanishes within numerical accuracy when $\hbar\omega(c^\dagger c + d^\dagger d)$ is ignored, as shown in Fig. SI.7(a). The full degeneracy of the OAM states does not allow for changes in the kinetic energy for motion along the z-direction, even if transitions between OAM states occur. Because the coupling between linear momentum and OAM involves a change in the kinetic energy, the energy and momentum conservation conditions described in the main text cannot be satisfied simultaneously. As a result, all OAM transitions are suppressed in the weak-coupling regime, leading to $\Delta T \approx 0$.

In the strong-coupling regime ($\beta = \beta_{\mathrm{DNA}}$), even if the OAM energy term $\hbar\omega(c^\dagger c + d^\dagger d)$ is disregarded, transitions between OAM states can occur. However, when the OAM states are fully degenerate, the kinetic energy along z may not increase during OAM transitions. In this case, the electron is expected to be almost completely reflected by the potential barrier when its initial kinetic energy, $\mathrm{KE}_0 = 1\,\mathrm{eV}$, is lower than the barrier height V_0. This prediction is

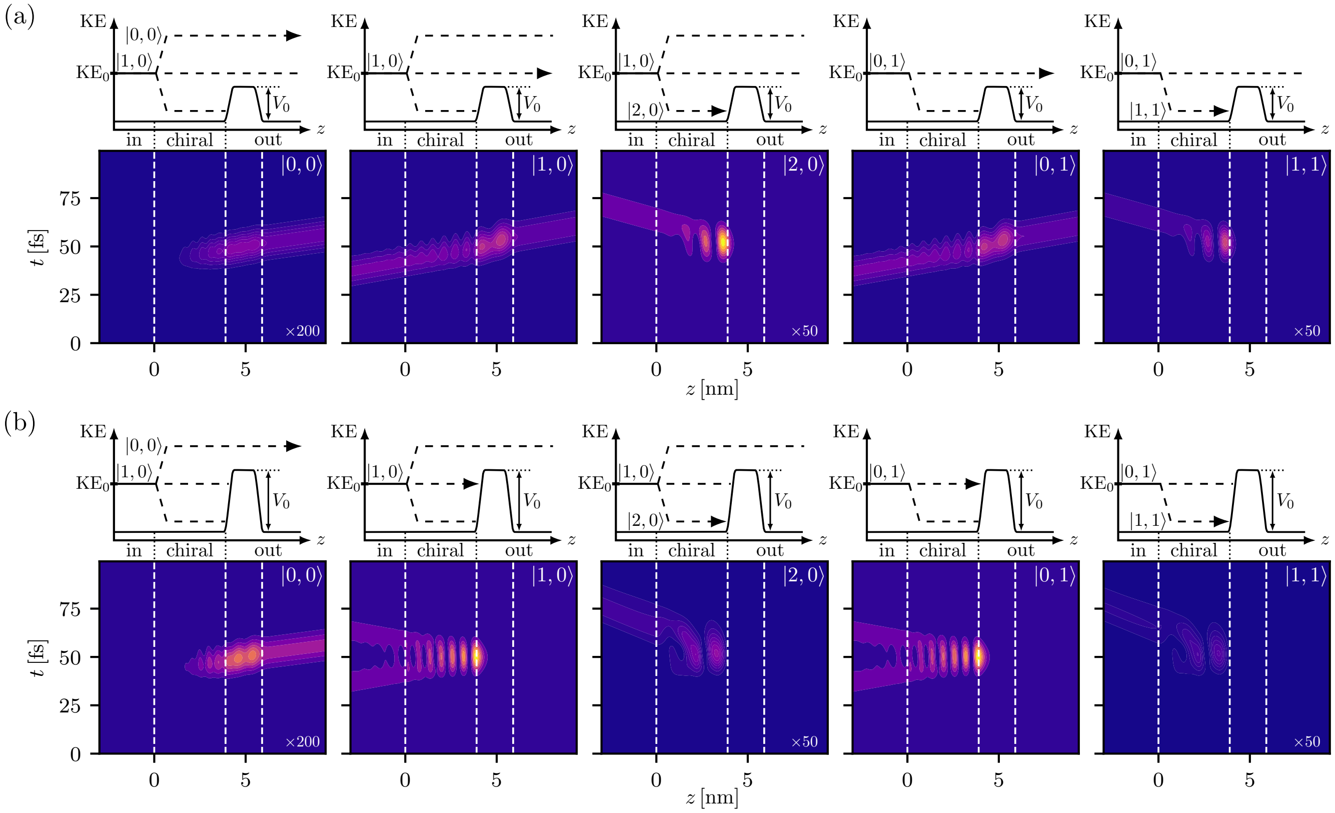

FIG. SI.1. Transient population dynamics of the electron wave packet conditioned on the OAM states $|n_c, n_d\rangle$, as a function of time t and coordinate z, for the settings corresponding to those used in Fig. 2 of the main text, where the initial OAM state is either $|1,0\rangle$ (the three leftmost columns) or $|0,1\rangle$ (the remaining columns). Each panel displays the population dynamics of a specific OAM component at position z, defined as $|\langle n_c, n_d, z|\psi(t)\rangle|^2$, where $|\psi(t)\rangle$ denotes the full electron state at time t, evolved under the Hamiltonian H' in the polaron picture. In (a) and (b), we show the cases $(V_0, \hbar\omega) = (0.7, 0.59)\,\mathrm{eV}$ and $(V_0, \hbar\omega) = (1.2, 0.85)\,\mathrm{eV}$, respectively, which correspond to the minima and maxima of the transmittance difference ΔT in Fig. 2(a), where the energy and momentum conservation conditions discussed in the main text are satisfied. In simulations, we assume that the z-dependence of the initial states is Gaussian; $\psi_{\mathrm{initial}} \propto \mathrm{e}^{-(z-z_0)^2/(2\Delta_z^2)+ik_0 z}$, with kinetic energy $\mathrm{KE}_0 = (\hbar k_0)^2/2m_e = 1\,\mathrm{eV}$ and the width $\Delta_z = 4\,\mathrm{nm}$. The center position z_0 is chosen so that the initial wave packet is well localized in the input region.

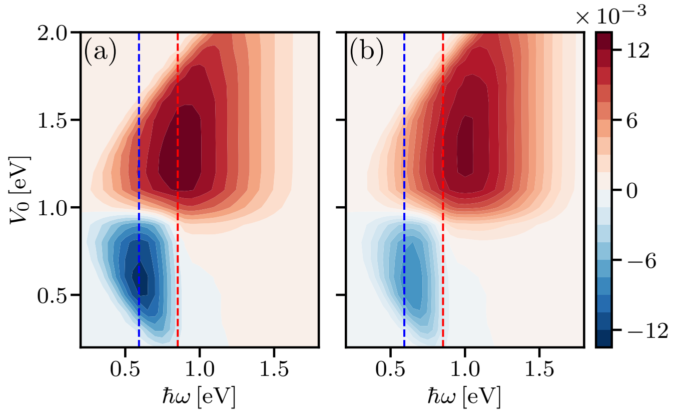

FIG. SI.2. (a) ΔT when $\beta = (R/2P)\sqrt{\hbar m_e \omega_0}$, with fixed $\hbar\omega_0 = 1\,\mathrm{eV}$. Vertical dashed lines indicate $\hbar\omega \in \{0.59, 0.85\}\,\mathrm{eV}$, where a minimum and a maximum in ΔT are expected to occur according to energy conservation and the linear momentum shift of $\hbar/P$ induced by the chiral coupling, as discussed in the main text. (b) ΔT when $\beta = (R/2P)\sqrt{\hbar m_e \omega}$, as in Fig. 2(a), demonstrating that the ω-dependence of β shifts the positions of the ΔT extrema.

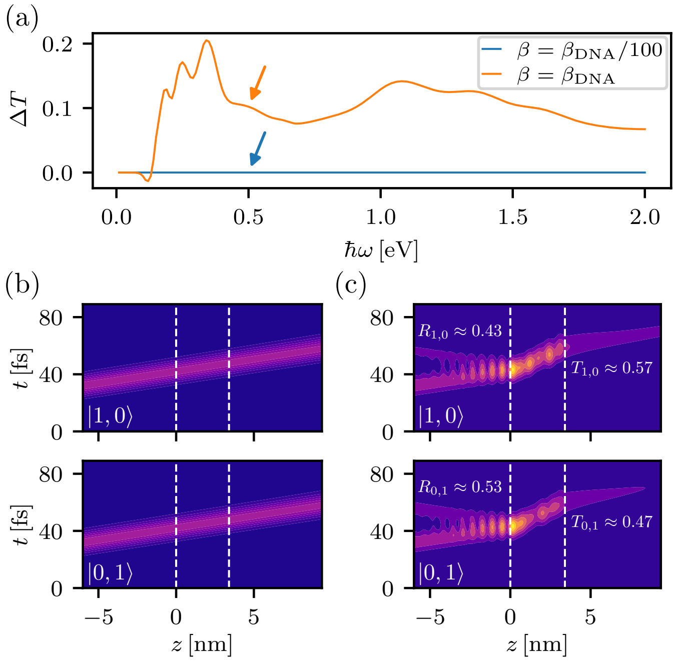

FIG. SI.3. (a) ΔT in the absence of a potential barrier ($V_0 = 0$, see Figs. 2 and 3(a) in the main text) for the weak-coupling ($\beta = \beta_{\mathrm{DNA}}/100$) and strong-coupling ($\beta = \beta_{\mathrm{DNA}}$) regimes. (b,c) The transient dynamics of the electron wave packet are shown as functions of time t and coordinate z for (b) the weak-coupling and (c) the strong-coupling cases, with $\hbar\omega = 0.5\,\mathrm{eV}$ marked by arrows in (a). Only in the strong-coupling case does notable reflection occur at the interface between the input and chiral regions, depending on the initial OAM state $|1,0\rangle$ or $|0,1\rangle$. The reflection probabilities for the initial OAM states, computed at a fixed time immediately after reflection, are $R_{1,0} \approx 0.43$ and $R_{0,1} \approx 0.53$. When summed with the final transmission probabilities $T_{1,0} \approx 0.57$ and $T_{0,1} \approx 0.47$, the results are close to unity, indicating that reflection primarily occurs at the interface between the input and chiral regions.

consistent with the simulated results in Fig. SI.7(b), where omitting $\hbar\omega(c^\dagger c + d^\dagger d)$ leads to negligible transmission probabilities for $V_0 > 1\,\mathrm{eV}$, resulting in $\Delta T \approx 0$. This is in contrast to the full model results shown in Fig. 3(a) of the main text, where significant ΔT is observed for $V_0 > 1\,\mathrm{eV}$.

Choice of parameters – Another significant difference between our work and that reported in Refs. [1, 2] is that, once the effective three-dimensional potential is specified, the continuum model can be simulated without introducing an additional tight-binding parameterization of hopping amplitudes or onsite orbital energies. The transport behavior is

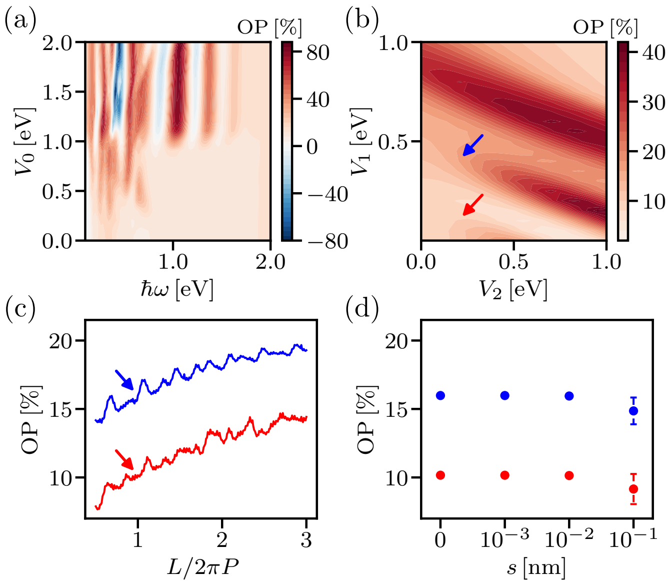

FIG. SI.4. Orbital polarization, defined as $\mathrm{OP} = (T_{1,0} - T_{0,1})/(T_{1,0} + T_{0,1})$, for the results shown in Fig. 3 of the main text.

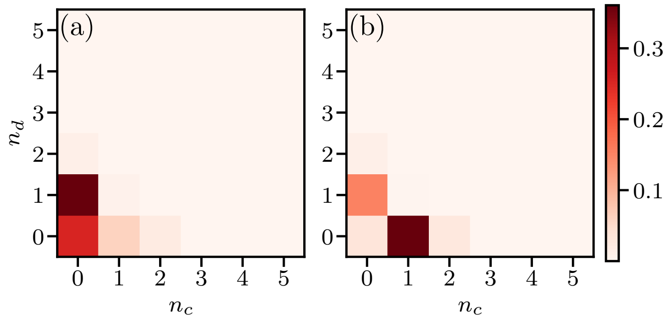

FIG. SI.5. For the initial OAM states (a) $|1, 0\rangle$ and (b) $|0, 1\rangle$ considered in Figure 3a of the main text, the OAM spectra of the electron wave packets transmitted through the chiral potential are shown. Here, the potential barrier after the chiral region is absent, i.e., $\hbar\omega = 1.0\,\mathrm{eV}$ and $V_0 = 0$. In (a), a high population of the $|0, 0\rangle$ state is observed, indicating that the $|1, 0\rangle \rightarrow |0, 0\rangle$ transition favorably occurs within the chiral region. In contrast, in (b), the population of the $|0, 0\rangle$ state is negligible because the $|0, 1\rangle \rightarrow |0, 0\rangle$ transition is weak. These OAM spectra demonstrate that the transition to $|0, 0\rangle$ is much more probable for the initial state $|1, 0\rangle$. When a potential barrier is introduced after the chiral region, the $|1, 0\rangle \rightarrow |0, 0\rangle$ transition provides sufficient longitudinal kinetic energy for the electron to overcome the barrier, thereby rationalizing the positive transmittance difference, $\Delta T > 0$, observed in the simulations for $\hbar\omega = 1.0\,\mathrm{eV}$ and $V_0 > 1.0\,\mathrm{eV}$ (see Figure 3a of the main text).

then determined by the geometric, confinement, barrier, and disorder parameters used to define the effective scattering problem.

Electronic dephasing noise – Our continuous-variable 3D model does not require any electronic dephasing to observe the CIOS effects present in our work. In contrast, tight-binding models often require dephasing noise to observe CIOS/CISS effects.

Access to transient dynamics – We also note that our work employs a wave-packet method, which enables monitoring of transient electron dynamics at the interfaces between the input/output and chiral regions, as well as within the chiral region itself (see Figs. SI.1 and SI.3). This wave-packet approach helps clarify the underlying CIOS mechanisms behind the simulated results and contrasts with the non-equilibrium Green's function method employed in Refs. [1, 2], which computes transmittance but does not provide access to transient dynamics.

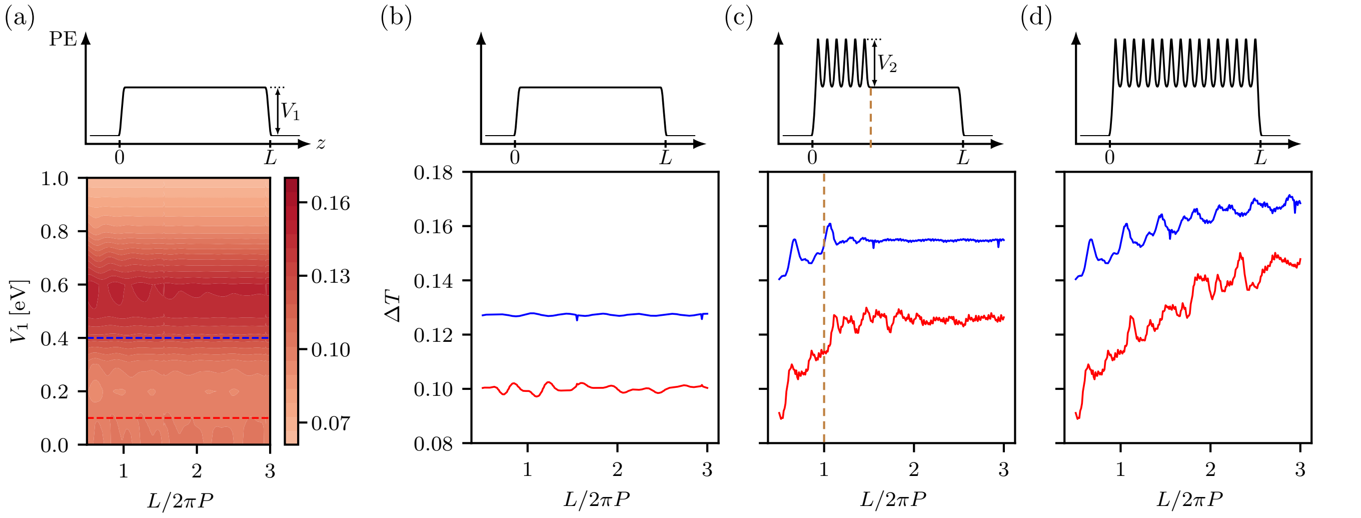

FIG. SI.6. (a) ΔT as a function of the length L and the potential offset V_1 of the chiral region without multiple Gaussian potentials ($V_2 = 0$, see Figs. 3(b) and (c) in the main text), showing negligible length dependence. (b) ΔT as a function of L for two representative offsets, $V_1 \in \{0.1, 0.4\}$ eV, marked by red and blue dashed lines in (a). (c) ΔT when multiple Gaussian potentials with $V_2 = 0.2$ eV are introduced up to the first chiral turn, producing notable length dependence up to $L \lesssim 2\pi P$ and negligible dependence beyond that. (d) ΔT when multiple Gaussian potentials with $V_2 = 0.2$ eV are present throughout the entire chiral region, resulting in notable length dependence over one to three chiral turns. These results indicate that the length dependence is strongly associated with the presence of multiple Gaussian potentials in the chiral region. We note that electronic dephasing is not required to observe the length dependence in our model.

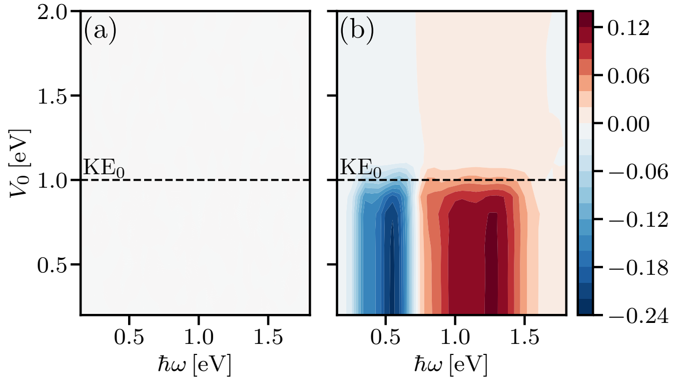

FIG. SI.7. ΔT when the OAM energy term $\hbar\omega(c^\dagger c + d^\dagger d)$ is omitted in (a) the weak-coupling ($\beta = \beta_{\mathrm{DNA}}/100$) and (b) the strong-coupling ($\beta = \beta_{\mathrm{DNA}}$) regimes (see Figs. 2 and 3(a) in the main text).

III. ANHARMONIC CONTINUOUS-VARIABLE MODELS

In the main text, we consider a continuous-variable 3D model in which the electron's confinement in the transverse (x, y) directions is described by a 2D harmonic potential. Here, we show that the CIOS effects remain robust even when the confinement potentials are anharmonic.

In Fig. SI.8, we consider the following anharmonic potentials in the chiral region ($0 \le z \le 2\pi P$):

$$\text{Quartic}: \quad W_Q(x,y,z) = v_Q((x - R\cos(z/P))^4 + (y - R\sin(z/P))^4), \tag{SI.7}$$

$$\text{Gaussian}: \quad W_G(x,y,z) = v_G(1 - \mathrm{e}^{-((x-R\cos(z/P))^2+(y-R\sin(z/P))^2)/2\sigma_G^2}), \tag{SI.8}$$

$$\text{Morse}: \quad W_M(x,y,z) = v_M((1 - \mathrm{e}^{-\alpha_M(x\cos(z/P)+y\sin(z/P)-R)})^2 + (1 - \mathrm{e}^{-\alpha_M(-x\sin(z/P)+y\cos(z/P))})^2). \tag{SI.9}$$

For the input and output regions, we consider the anharmonic potentials whose equilibrium positions are independent of z, by fixing the value of z to 0 for $z < 0$ and $2\pi P$ for $z > 2\pi P$ in Eqs. (SI.7)-(SI.9). This results in straight

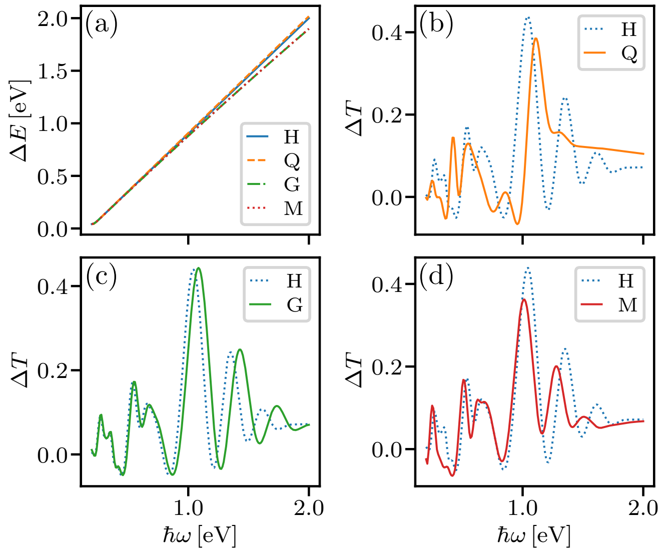

FIG. SI.8. (a) Energy gap ΔE between the ground and the first excited states of the harmonic (H), quartic (Q), Gaussian (G) and Morse (M) potentials in the xy-plane at fixed z, shown as a function of ω. (b-d) Transmittance difference for initial states carrying OAM of $\hbar$ or $-\hbar$. See the SI text for further details.

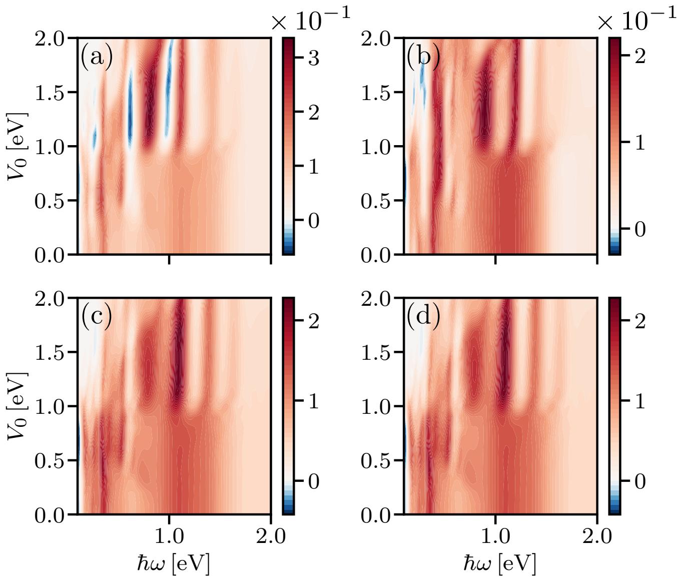

FIG. SI.9. In the main text, the transmittance difference between the two initial OAM states $|1,0\rangle$ and $|0,1\rangle$ is considered. Here, using the same model parameters as in Figure 3a of the main text, we examine additional pairs of initial OAM states and show their transmittance differences: (a) $|2,1\rangle$ and $|1,2\rangle$; (b) $|3,2\rangle$ and $|2,3\rangle$; (c) random mixtures of $|1,0\rangle$, $|2,1\rangle$, and $|3,2\rangle$, and of $|0,1\rangle$, $|1,2\rangle$, and $|2,3\rangle$; and (d) the coherent superpositions $3^{-1/2}(|1,0\rangle+|2,1\rangle+|3,2\rangle)$ and $3^{-1/2}(|0,1\rangle+|1,2\rangle+|2,3\rangle)$. The initial OAM states $|1,0\rangle$, $|2,1\rangle$, and $|3,2\rangle$ all carry an OAM of $\hbar$ about the z-axis, whereas $|0,1\rangle$, $|1,2\rangle$, and $|2,3\rangle$ carry an OAM of $-\hbar$ about the z-axis. The initial longitudinal kinetic energy along the z-direction is set to 1.0 eV, independent of the initial OAM state. The transmittance differences shown in (c) and (d) are nearly identical, indicating that coherence between the initial OAM states does not play a significant role in the scattering process within the chiral region.

waveguides smoothly connected to the chiral waveguides, as in the harmonic case considered in the main text. We express the parameters of each anharmonic potential as functions of ω from the harmonic case, namely, $v_Q = m_e^2\omega^3/5\hbar$, $v_G/\sigma_G^2 = m_e\omega^2$ and $v_M\alpha_M^2 = m_e\omega^2/2$, so that the energy-gap ΔE between the ground and the first excited states of the anharmonic potential is approximately equal to the energy quanta $\hbar\omega$ of the harmonic case, as shown in Fig. SI.8(a).

As in Fig. 3(a) of the main text, we consider a rectangular potential barrier located after the chiral region with a fixed potential height of $V_0 = 1.2\,\mathrm{eV}$. We assume that the initial state, localized in the input region, is a superposition of the two degenerate first excited states of the anharmonic potential with a relative phase of i or $-i$, similar to $|0,1\rangle \pm i\,|1,0\rangle$ in the harmonic case, multiplied by a Gaussian wave packet as a function of z, carrying the initial kinetic energy of $\mathrm{KE}_0 = 1\,\mathrm{eV}$. These initial states carry OAM of approximately $\hbar$ or $-\hbar$, which is conserved within the input region until the wave packet enters the chiral region. The dissociation energies of the Gaussian and Morse potentials are fixed to $v_{\mathrm{G}} = v_{\mathrm{M}} = 20\,\mathrm{eV}$, ensuring that the electron remains confined within the potentials. In Figs. SI.8(b)-(d), the transmittance difference between the two initial states is shown, indicating that the orbital selectivity of the harmonic and anharmonic models is qualitatively similar. These results demonstrate that the CIOS effects are robust against variations in the confinement potentials.

IV. FINITE BIAS AND TIME-DEPENDENT POTENTIALS

In the main text, we consider the zero-bias case, with no potential energy difference between the input and output regions. In addition, we assume time-independent potentials in the chiral region, under which electron energy is conserved. Here, we extend the analysis to two additional scenarios. First, we introduce a finite bias by increasing (decreasing) the potential energy of the input (output) region. Second, we consider time-dependent potentials within the chiral region, so that electron energy is no longer conserved. In both cases, we find that the CIOS effect remains robust.

In Fig. SI.10(a), the potential energy of the input (output) region is increased (decreased) by $V_3/2$, resulting in a finite bias of V_3. The time-independent Gaussian potentials with $L = 2\pi P$, used in Fig. 3 of the main text, are assumed in the chiral region. For the two representative cases $V_1 \in \{0.1, 0.4\}\,\mathrm{eV}$ and $V_2 = 0.2\,\mathrm{eV}$ considered in Fig. 3 of the main text, Fig. SI.10(a) shows the transmittance difference ΔT as a function of the finite bias V_3. The initial kinetic energy of the electron wave packet is taken to be $\mathrm{KE}_0 = 1\,\mathrm{eV}$, so that the total energy becomes $\mathrm{KE}_0 + V_3/2$. Notably, the transmittance difference ΔT remains on the order of 0.1 over a broad range of the bias, $V_3 \in [0, 2]\,\mathrm{eV}$, indicating that the CIOS effect is robust in the presence of the finite bias.

In Figs. SI.10(b)-(d), the zero-bias case ($V_3 = 0$) is considered with time-dependent Gaussian potentials in the chiral region. The offset is taken as $V_1 \in \{0.1, 0.4\}\,\mathrm{eV}$, with the corresponding results shown in red and blue, respectively. The amplitude of the Gaussian potential centered at position z is assumed to be time-dependent and modeled as $V_2 + \sum_{j=1}^{3} A_j \sin(k_j z + \omega_j t + \phi_j)$ with $V_2 = 0.2\,\mathrm{eV}$. For each simulation, the amplitudes A_j of the fluctuating terms are randomly drawn from independent normal distributions with zero mean and standard deviation σ_A. Similarly, the phases ϕ_j are randomly drawn from uniform distributions over $[0, 2\pi]$. The wave vectors are taken as $k_j = j/P = 2\pi j/L$, with dispersion relations $\omega_j = \mu k_j$, where μ sets the time scale of the fluctuating potentials. Figures SI.10(b)-(d) show statistical data obtained from simulations with the randomly generated amplitudes A_j and phases ϕ_j.

In Fig. SI.10(b), the distributions of the kinetic energy of the electron wave packet in the output region, after scattering within the chiral region, are shown as a function of the parameter μ in the dispersion relations, with fixed $\sigma_A = 0.2\,\mathrm{eV}$. For a detailed kinetic energy analysis, here we consider a one-dimensional model without orbital degrees of freedom. Note that, due to the time-dependent potentials in the chiral region, the average kinetic energy of the output electron wave packet differs from the initial kinetic energy $\mathrm{KE}_0 = 1\,\mathrm{eV}$. We consider $\mu \in \{0.2, 0.5\}\,\mathrm{nm/fs}$ as two representative cases, and provide further analysis in Figs. SI.10(c) and (d), respectively.

In Fig. SI.10(c), for $\mu = 0.2\,\mathrm{nm/fs}$, the kinetic energy of the output electron is shown as a function of σ_A (top panel, 1D model), indicating that electron energy is not conserved over the σ_A range considered in the simulations. For the 3D model including orbital degrees of freedom, the transmittance difference ΔT (middle panel) and orbital polarization (bottom panel) are shown as functions of σ_A, demonstrating that the CIOS effect remains robust under the time-dependent potentials. Similar results are obtained for $\mu = 0.5\,\mathrm{nm/fs}$, as shown in Fig. SI.10(d).

V. ORBITAL TO SPIN SELECTIVITY

In the main text, we discuss that orbital selectivity can give rise to spin selectivity when spin-OAM states are correlated before transmission to the chiral region, even if the spin-orbit interaction within the chiral region is negligible. Here we provide an example by considering a finite input region with spin-orbit coupling (SOC) for $z_1 \leq z \leq z_2$, as schematically shown in Fig. SI.11(a). The SOC is modeled by

$$H_{\mathrm{SOC}} = \alpha_0 \boldsymbol{\sigma} \cdot (\nabla W \times \mathbf{p}) \Theta(z - z_1) \Theta(z_2 - z), \tag{SI.10}$$

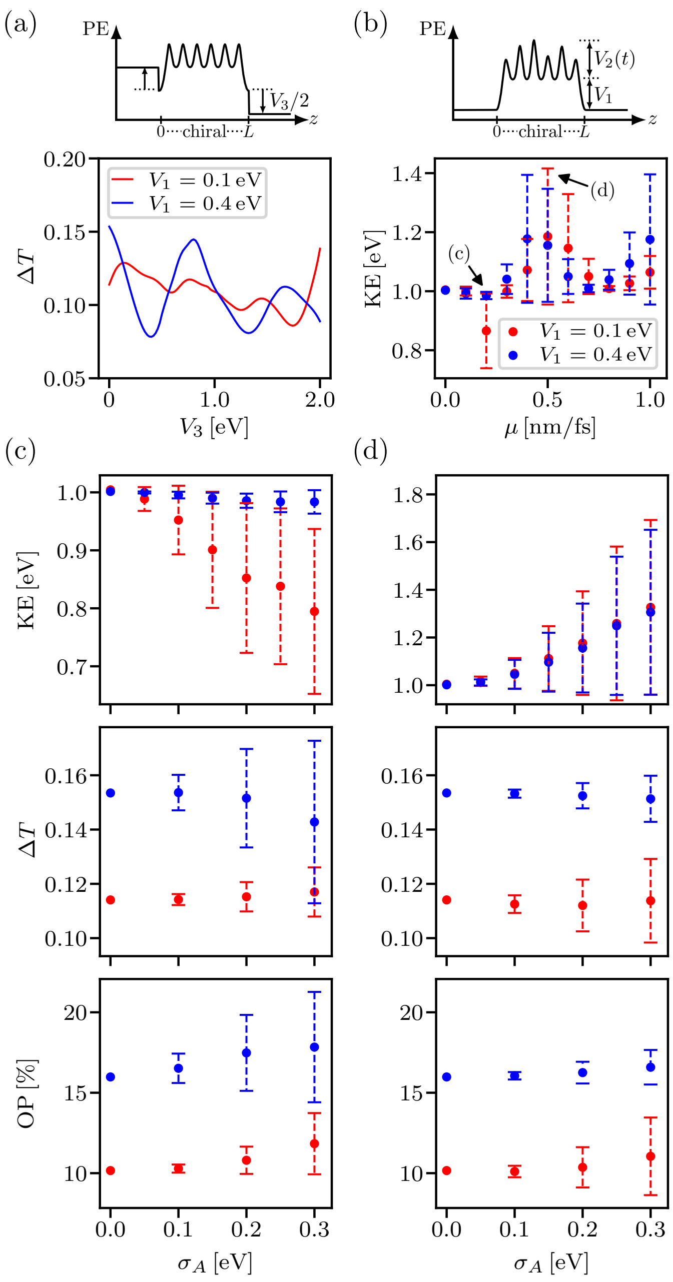

FIG. SI.10. (a) ΔT as a function of the bias V_3, for the time-independent Gaussian potential model from Fig. 3 of the main text, with $V_1 \in \{0.1, 0.4\}$ eV and $V_2 = 0.2$ eV. (b) The kinetic energy of the output electron in the 1D model (no orbital degrees of freedom) as a function of μ in the dispersion relations of the time-dependent Gaussian potential model (see SI text). For (c) $\mu = 0.2$ nm/fs and (d) $\mu = 0.5$ nm/fs, the kinetic energy of the output electron (top panel, 1D model), the transmittance difference ΔT (middle panel, 3D model), and the orbital polarization (bottom panel, 3D model) are shown.

where W is the harmonic potential energy within the achiral input region, where equilibrium positions are independent of z, and $\Theta(z)$ is the step function defined as $\Theta(z) = 1$ for $z > 0$ and $\Theta(z) = 0$ otherwise. For simplicity, we assume that the length of the SOC region is $z_2 - z_1 = 1$ nm and $\hbar\alpha_0 = 0.01\,\mathrm{nm}^2$, so that the spin-energy splitting scales as $\hbar\alpha_0 m\omega^2 \in \{0.005, 0.5\}$ eV for $\hbar\omega \in \{0.2, 2\}$ eV. In the simulations, we consider a random mixture of spin up and down states at the initial time, where an electron is localized in the input region without SOC (i.e., $z < z_1$) and carries zero OAM but has linear momentum along the z-direction, with the initial kinetic energy of $\mathrm{KE}_0 = 1\,\mathrm{eV} + \hbar\omega$. The additional energy $\hbar\omega$ is introduced so that, when the OAM becomes $\hbar$ or $-\hbar$ via the SOC, the kinetic energy along the propagation z-direction is reduced to 1 eV, consistent with the simulation settings in the main text.

As the electron wave packet propagates through the SOC region, the population of the initial angular momentum

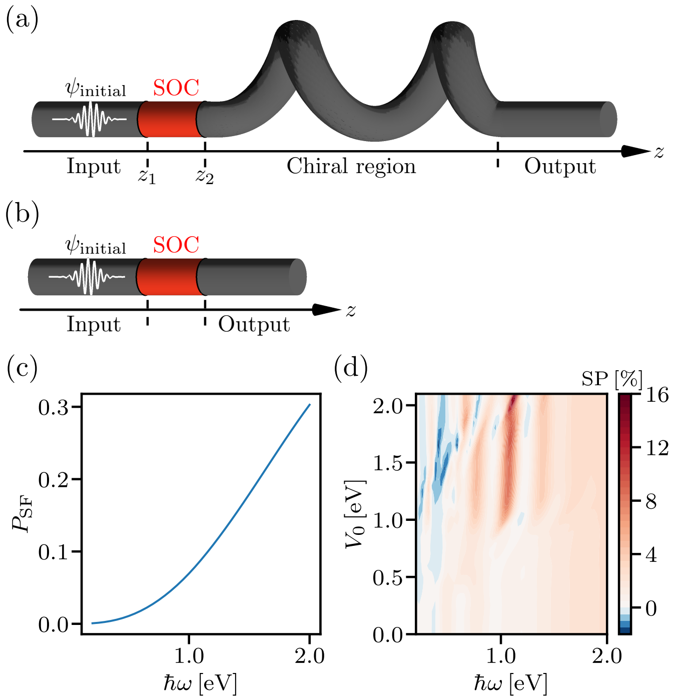

FIG. SI.11. (a) Schematic representation of a 3D model for electron transfer through input-chiral-output regions, where SOC is present in the input region for $z_1 < z < z_2$. (b) Simplified setup without the chiral region. (c) Population of spin-flipped states transmitted through the SOC region, when the chiral region is omitted (see (b)). (d) Spin polarization of the full model in the presence of the chiral region with zero SOC (see (a)).

states, namely, $|n_c = 0, n_d = 0, \uparrow\rangle$ or $|n_c = 0, n_d = 0, \downarrow\rangle$, is converted into that of the spin-flipped angular momentum states, $|n_c = 1, n_d = 0, \downarrow\rangle$ or $|n_c = 0, n_d = 1, \uparrow\rangle$, while conserving the z-component of total angular momentum. Here, the net spin polarization is zero as the SOC region is achiral. However, the spin-OAM states are correlated, as $|n_c = 0, n_d = 0, \uparrow\rangle$ and $|n_c = 1, n_d = 0, \downarrow\rangle$ (or $|n_c = 0, n_d = 0, \downarrow\rangle$ and $|n_c = 0, n_d = 1, \uparrow\rangle$) are superposed via the SOC when the initial state carries spin up (or down). The other states carrying the same total angular momentum $\hbar/2$ (or $-\hbar/2$), such as $\{|n_c = N, n_d = N, \uparrow\rangle, |n_c = N+1, n_d = N, \downarrow\rangle\}$ (or $\{|n_c = N, n_d = N+1, \uparrow\rangle, |n_c = N, n_d = N, \downarrow\rangle\}$) with positive integer N, have negligible contributions to the electron dynamics in the input region, as the SOC considered in our simulations is not strong enough to significantly populate these states. The total angular momentum conservation arises as the Hamiltonian of the achiral input region is invariant under rotations about the z-axis, ensuring that the z-component of the total angular momentum remains constant until the electron enters the chiral region.

To quantify the population converted into the spin-flipped states, we omit the chiral region, as shown in Fig. SI.11(b), and examine the spin-flipped population P_{SF} as a function of ω, as shown in Fig. SI.11(c). When $\hbar\omega \approx 1\,\text{eV}$, the spin-flipped population is approximately 0.07. Although the spin-flipped population, along with the corresponding spin-OAM correlations, is small, it can nevertheless give rise to a notable spin selectivity. In Fig. SI.11(d), we consider the full setup, including the chiral region without SOC (see Fig. SI.11(a)) and a rectangular energy barrier of height V_0 located after the chiral region, and show the spin polarization (SP) of the transmitted electron wave. Notably, an SP of the order of 10 % is observed at $\hbar\omega \approx 1\,\text{eV}$. These results demonstrate that even if the chiral region has negligibly weak SOC, the orbital selectivity of the chiral region can give rise to spin selectivity when the spin-OAM states are correlated prior to entering the chiral region, e.g., via relatively strong spin-orbit interaction within metallic electrodes.

VI. NUMERICAL METHODS AND IMPLEMENTATION

To implement numerical simulations of the three-dimensional continuous-variable models considered in this work, we discretized the z coordinate and approximated derivatives with respect to z, appearing in the momentum and kinetic operators, using a fourth-order finite-difference method. The grid spacing in z was systematically reduced until convergence of the simulation results was achieved. To reduce the total number of discretized z points, we introduced absorptive layers at the ends of the input and output regions. These were implemented using an imaginary term

added to the Hamiltonian

$$\Gamma_a(z) = -i\gamma_a(1 - e^{-(z-z_0)^2/2\sigma_a^2})/2, \quad \text{(SI.11)}$$

where z_0 denotes the z-coordinate of the open end of the input or output region. The parameters γ_a and σ_a were chosen such that dissipation of the electron wave packet by the imaginary term is negligible until the initial electron wave packet is scattered from the chiral region, while wave packets reflected from or transmitted through the chiral region are efficiently absorbed, ensuring that re-entry of these wave packets into the chiral region is negligible. For example, in the simulations shown in Fig. 3, we used 40 discretized z points per nm, corresponding to a total of 3,736 discretized z points over the input, chiral, and output regions. For the transverse motion of the electron in the xy-plane, described in the eigenbasis $|n_c, n_d\rangle$ of the harmonic oscillators, we considered 10 levels in each direction, namely $n_c, n_d \in \{0, 1, \cdots, 9\}$, to obtain numerical convergence of the simulation results in Fig. 3, leading to a total dimension of 373,600. Comparable or even higher dimensions were considered in the simulations shown in other figures.

We computed the time evolution of the electron wave packet using a fourth-order Runge-Kutta method with a time step of $dt = 0.002\,\text{fs}$ to achieve convergence of the simulation results. The transmittance T_{n_c,n_d} was obtained by computing the population dissipated by the absorptive layer in the output region.

The lattice-like potentials considered in Fig. 3 and Fig. SI.10 were multiplied by a smooth step function to ensure a smooth transition between input, chiral and output regions, defined as

$$f(z; z_0, z_1) = \begin{cases} 192(z - z_0 + 0.5)^5 - 240(z - z_0 + 0.5)^4 + 80(z - z_0 + 0.5)^3, & z_0 - 0.5 < z < z_0 \\ 1, & z_0 \le z \le z_1 \\ 1 - (192(z - z_1)^5 - 240(z - z_1)^4 + 80(z - z_1)^3), & z_1 < z < z_1 + 0.5 \end{cases}$$

for a chiral region $z_0 < z < z_1$.

The static disorder in Fig. 3(d) and dynamic disorder in Fig. SI.10 were implemented by running multiple simulations with randomly sampled parameters, followed by ensemble averaging. For each case, we used 200 and 1000 realizations, respectively.

[1] Gersten, J.; Kaasbjerg, K.; Nitzan, A. Induced spin filtering in electron transmission through chiral molecular layers adsorbed on metals with strong spin-orbit coupling. *The Journal of Chemical Physics* **2013**, *139*, 114111.

[2] Liu, Y.; Xiao, J.; Koo, J.; Yan, B. Chirality-driven topological electronic structure of DNA-like materials. *Nature Materials* **2021**, *20*, 638–644.
